# Association between age-related macular degeneration and 25(OH) vitamin D levels in the Turkish population

**DOI:** 10.5935/0004-2749.20220002

**Published:** 2022

**Authors:** Naciye Kabataş, Aysun Şanal Doğan, Mevlüt Yılmaz, Emrah Utku Kabataş, Tolga Biçer, Sinan Çalışkan, Osman Çelikay, Fatma Uçar, Canan Gürdal

**Affiliations:** 1 Department of Ophtalmology, Dışkapı Yıldırım Beyazıt Research and Education Hospital, Ankara, Turkey; 2 Department of Medicinal Chemistry , Dışkapı Yıldırım Beyazıt Research and Education Hospital, Ankara, Turkey

**Keywords:** Choroid neovascular membrane, Macular dege neration, Optical coherence Tomography, Fibrosis, Retina, Vitamin D, Vitamin D deficiency, Membrana neovascular da coroide, Degeneração macular, Tomografia de coerência óptica, Fibrose, Retina, Vitamina D, Deficiência de vitamina D

## Abstract

**Purpose:**

Age-related macular degeneration is the most common cause of blindness in developed countries, and several factors have been attributed for its etiology. This study was conducted to explore the relationship between serum vitamin D levels and age-related macular degeneration.

**Methods:**

We retrospectively analyzed the data of 114 patients with age-related macular degeneration. A total of 102 patients who did not have any other diseases than refractive error were allocated to the control group. The best-corrected visual acuity, fundus findings, and spectral domain optical coherence tomography findings were analyzed. Patients were allocated to groups based on the Age-related Eye Disease Study classification. Serum 25(OH) vitamin D levels were measured. The central foveal thickness and the subfoveal choroidal thickness were measured by optical coherence tomography.

**Results:**

The 25(OH) vitamin D levels in ageand gender-matched patients with age-related macular degeneration and in healthy subjects were 14.6 ± 9.8 and 29.14 ± 15.1 ng/ml, respectively. The age-related macular degeneration group had significantly lower vitamin D levels than the control group (p>0.001). The subfoveal choroidal thickness was lower in patients with age-related macular degeneration (p>0.001). The 25(OH) vitamin D level showed a weak positive correlation with choroidal thickness (*r*=0.357, p=0.01). When the level of 25(OH) vitamin D was evaluated according to the stages of age-related macular degeneration, it was found to be lower in the advanced-stage disease (p=0.01). The risk for the development of choroid neovascular membrane and subretinal fibrosis was found to increase with decreased vitamin D levels.

**Conclusions:**

Significantly decreased levels of 25(OH) vitamin D in advanced-stage age-related macular degeneration suggest a significant correlation existing between vitamin D deficiency and age-related macular degeneration development. Further studies are required to examine whether vitamin D supplementation has an effect on the development and progression of age-related macular degeneration.

## Abstract (pt)

**Objetivo:**

A degeneração macular relacionada à idade é a causa mais comum de cegueira em países desenvolvidos e muitos fatores etiológicos têm-lhe sido atribuídos. O objetivo do presente estudo foi investigar a relação entre os níveis séricos de vitamina D e a degeneração macular relacionada à idade.

**Métodos:**

Os dados de 114 pacientes com degeneração macular relacionada à idade foram analisados retrospectivamente. Foram alocados no Grupo Controle 102 pacientes sem registro de outras doenças além do erro refrativo. A acuidade visual melhor corrigida, os achados do exame de fundo de olho e os da tomografia de coerência óptica de domínio espectral foram analisados. Os pacientes foram alocados em grupos de acordo com a classificação do *Age-Related Eye Disease Study* (Estudo da Doença Ocular Relacionada à Idade). Os níveis séricos de vitamina D 25(OH) foram medidos. A espessura foveal central e a espessura da coroide subfoveal foram medidas com tomografia de coerência óptica.

**Resultados:**

Os níveis de vitamina D 25(OH) em pacientes com degeneração macular relacionada à idade e em indivíduos saudáveis pareados por idade e sexo foram 14,6 ± 9,8 ng/mL e 29,14 ± 15,1 ng/mL, respectivamente. Os níveis de vitamina D foram significativamente menores no Grupo da Degeneração Macular relacionada à idade em comparação com o Grupo Controle (p>0,001). O valor da espessura da coroide subfoveal foi menor em pacientes com degeneração macular relacionada à idade (p>0,001). Foi encontrada uma fraca correlação positiva entre o nível de vitamina D 25(OH) e a espessura da coroide (r=0,357, p=0,01). O nível de vitamina D 25(OH), quando avaliado de acordo com os estágios da degeneração macular relacionada à idade, revelou ser menor na doença em estágio avançado (p=0,01). Constatou-se um risco aumentado de desenvolvimento de membrana neovascular da coroide e de fibrose sub-retiniana com a diminuição dos níveis de vitamina D.

**Conclusões:**

A diminuição significativa dos níveis de vitamina D 25(OH) na degeneração macular relacionada à idade em estágio avançado sugere a presença de uma correlação significativa entre a deficiência de vitamina D e o desenvolvimento dessa patologia. Mais estudos são necessários para investigar se a suplementação de vitamina D tem ou não influência no desenvolvimento e progressão da degeneração macular relacionada à idade.

## INTRODUCTION

Age-related macular degeneration (ARMD) is the most common cause of central visual loss among individuals with advanced age in developed countries^(1,2)^. Although several factors, including genetic, nutritional, and environmental, and increased inflammation and neovascularization have been attributed for the etiology of ARMD, the etiology has not been completely explained^(3)^.

The biologically active form of vitamin D 1,25(OH)2 vitamin D is a steroid hormone and has been demonstrated to possess anti-inflammatory, antiangiogenic, and anticarcinogenic properties, besides preserving calcium and phosphorous homeostasis and contributing to development of healthy bones^(4)^. Studies have also shown that vitamin D protects the cellular membrane and proteins against oxidative damage^(4-6)^ and protects the nervous system cells by regulating the release of neuron growth factor (NGF)^(5,6)^. It has also been reported that vitamin D is necessary for the maintenance of healthy functions in several organs and tissues and exerts protective effects against aging^(7)^.

Animal studies have shown that vitamin D supplementation reduces retinal inflammation by causing a significant decrease in macrophage count and accumulation of intracellular beta-amyloid as an aging marker in retinal cells^(7)^. These findings suggest that vitamin D could play a role in the maintenance of healthy retinal functions and protection of the retina against aging. It is suggested that vitamin D deficiency causes aging and degeneration in the retina due to these effects.

Studies also suggest an association between vitamin D levels and ARMD development; however, there is a lack of a sufficient number of studies on this issue, and the results are also conflicting. Therefore, the present study was conducted to investigate whether serum vitamin D levels have an effect on the prevalence and severity of ARMD.

## METHODS

This study was conducted at the Retina Division of the Ophthalmology Clinic of Ankara Dışkapı Yıldırım Beyazıt Research and Training Hospital according to the principles of the Declaration of Helsinki after obtaining approval from the local ethics committee. All participants provided written informed consents. We conducted a retrospective analysis of the vitamin D levels of 114 patients who had been diagnosed with ARMD and actively followed up at our clinic. A total of 102 ageand sex-matched patients who did not have any ocular or systemic diseases other than refractive error were allocated to the control group.

Patients with ARMD were classified according to the classification of the Age-related Eye Disease Study (AREDS) as follows:

- Group 1 (early-stage ARMD): Patients who had widespread small drusen, less than 20 moderate drusen, or pigment anomalies in at least one eye;
- Group 2 (intermediate-stage ARMD): Patients with large drusen, widespread moderate drusen, or non-central geographic atrophy in at least one eye;
- Group 3 (advanced-stage ARMD) included subjects who had a visual acuity (VA) lower than 20/32 due to early-stage ARMD lesions such as geographic atrophy involving the fovea or choroid neovascularization or non-drusen retinal pigment epithelium (RPE) detachment or subfoveal drusen in at least one eye.

If a patient had eyes with different stages of ARMD, we evaluated the eye that had advanced disease.

All patients were subjected to a complete ophthalmological examination, including best-corrected visual acuity (BCVA) assessment using the Snellen chart, intraocular pressure measurements, biomicroscopic examination, fundus examination, and spectral domain optical coherence tomography (SD-OCT, RTVue-XR 100 Avanti software v.6.1, Optovue, Inc., Fremont, CA, USA). Sun exposure (less than 1 h/day, 1-3 h/day, longer than 3 h/day), smoking status, and presence of diabetes mellitus (DM) and hypertension (HT) were examined.

The central foveal thickness and the subfoveal choroidal thickness were measured by OCT, and the presence of subretinal fibrosis and choroid neovascular membrane was recorded.

The following patients were excluded from the study: those who had been receiving immunomodulator treatment or who had chronic renal, hepatic, or parathyroid diseases or who had received intravitreal anti-VEGF injections during the recent 1 month or who were using antioxidant or vitamin D treatment.

### Blood sampling

Blood samples were collected in polypropylene tubes and immediately centrifuged at 1500× *g* for 10 min within 1.5 h after withdrawal. Total serum 25(OH) vitamin D levels were measured using an autoanalyzer (LIAISON DiaSorin, Italy) based on the principle of chemiluminescence immunoassay (CLIA) method according to manufacturer’s instructions. The laboratory levels of vitamin D are as follows: ≤20 ng/ml indicates deficiency, between 20 and 30 ng/ml indicates insufficient, and ≥30 ng/ml is sufficient.

### Statistical analysis

Data were evaluated using the Statistical Package for the Social Sciences (SPSS) v 20.0 program. Normality distribution of data was evaluated using the Kolmogorov-Smirnov test. Parametric variables were evaluated using Student’s *t*-test, and nonparametric variables were evaluated using the Mann-Whitney U test and Kruskal-Wallis test. Data were presented as mean ± standard deviation. Multivariate regression analysis was performed for measurement of the associations between independent variables. Results were evaluated at 95% confidence intervals, and a p value <0.95 was accepted as statistically significant.

## RESULTS

The female/male ratio was 64/50 in the ARMD group and 57/45 in the control group, with no statistically significant difference between the two groups (p=0.235). The mean age of subjects was 71.5 ± 7.9 years in the ARMD group and 69.4 ± 10.1 years in the control group, which was also not statistically significant different (p=0.148). The mean serum vitamin D levels were 14.4 ± 9.6 and 29.4 ± 14.6 ng/ml in the ARMD group and control group, with the difference being statistically significant (p<0.001). Patients in the ARMD group had a statistically significantly lower choroidal thickness than those in the control group (p<0.001). Table 1 shows the other parameters of the study groups.

**Table 1:** Demographic data of the patients

	Age related macular degeneration group (n=114)	Control group (n=102)	p-value
Body mass index	28.4 ± 3.5	27.9 ± 2.8	0.326
Diabetes mellitus, n (%)	30 (26.3)	22 (21.5)	0.465
Hypertension, n (%)	60 (52.6)	49 (48.0)	0.592
Smoking, n (%)	58 (50.9)	25 (24.5)	**0.001**
Central foveal thickness (micron)	266.7 ± 88.4	255.5 ± 26.6	0.209
Choroidal thickness (micron)	241.9 ± 115.5	324.4 ± 83.7	**<0.0001**
Visual acuity (logMAR)	0.6 ± 0.9	0.9 ± 0.1	**0.001**
Sun exposure (<1 h/day) n (%)	88 (77.2)	60 (58.8)	**0.01**

When the levels of 25(OH) vitamin D were compared according to the ARMD stages, they were found to be significantly lower in advanced-stage ARMD than in earlyand intermediate-stage ARMD (p<0.0001 and = 0.005, respectively). There was no significant difference between patients with earlyand intermediate-stage ARMD (p=0.45) (Table 2).

**Table 2:** Serum 25 (OH) vitamin D levels of the patients

	Serum 25(OH) vitamin D level (ng/ml)		p-value
Early age-related macular degeneration (n=46)	18.2 ± 11.9		Early-Intermediate p=0.45
Intermediate age-related macular degeneration (n=18)	15.6 ± 6.9		Early-Advanced p<0.0001
Advanced age-related macular degeneration (n=50)	10.9 ± 6.8		Intermediate-Advanced p=0.005

When ARMD development was considered as the dependent variable and DM, HT, smoking, sun exposure, body mass index (BMI), and 25(OH) vitamin D levels (when ≤20 ng/ml is defined as vitamin D deficiency and >20 ng/ml is not vitamin D deficiency) were considered as independent variables in the multivariate regression analysis, vitamin D deficiency (OR: 7.11; 95% CI, 3.3315.17; p<0.0001) and smoking (OR: 4.64, 95% CI, 1.9411.12, p=0.001) were found to be associated with ARMD.

Analysis of vitamin D levels in smokers and nonsmokers revealed lower levels in smokers (16.10 ± 8.8 vs 22.67 ± 15.85 ng/ml; p<0.0001).

Concerning the type of ARMD, the serum vitamin D levels were 11.4 ± 5.1 ng/ml in the wet-type ARMD and 15.3 ± 10.9 ng/ml in the dry-type ARMD, with the difference being statistically significant (p=0.01).

When choroid neovascular membrane development was considered as the dependent variable and DM, HT, smoking, sun exposure, BMI, and 25(OH) vitamin D levels (when ≤20 ng/ml is defined as vitamin D deficiency and >20 ng/ml is not vitamin D deficiency) were considered as independent variables in the multivariate regression analysis, only vitamin D deficiency was found to be associated with ARMD (OR: 6.33 95% CI, 1.32-30.25) (p=0.021).

The mean serum vitamin D level was 11.1 ± 6.3 ng/ml in patients with subretinal fibrosis, and it was found to be statistically significant when compared to without subretinal fibrosis (p<0.0001).

## DISCUSSION

This study demonstrated that serum vitamin D levels were significantly lower in patients with ARMD and also in patients with advanced-stage ARMD than in patients with earlyand intermediate-stage ARMD.

Vitamin D deficiency may be observed even in younger individuals in developed countries, and its prevalence increases with age (approximately 87% in the elderly)^(8)^. It has been suggested that vitamin D exerts a protective effect against ARMD by affecting various steps of ARMD physiopathology. For instance, vitamin D might control the release of various cytokines and exert anti-inflammatory activities and hence could prevent cell death and aging of the retina by reducing inflammation^(9)^. Moreover, vitamin D has been shown to possess antioxidant properties and protect structural proteins and DNA against oxidative damage^(10)^. Vitamin D plays an important role in the maintenance of healthy RPE functions by triggering autophagy in the degenerated RPE^(11)^. In addition to these effects, vitamin D has been reported to possess antiapoptotic and antiangiogenic properties and to be important in the maintenance of healthy retinal functions and in the prevention of degeneration^(11,12)^.

Earlier studies investigating the association between vitamin D levels and ARMD have yielded conflicting results. For instance, Parekh et al. detected a reduction in ARMD prevalence in subjects receiving vitamin D supplementation, whereas a comprehensive study conducted in Israel reported no association between vitamin D levels and ARMD. Moreover, Wu et al. did not find a relationship between vitamin D levels and ARMD types in a meta-analysis ^(13,14,15)^. In a larger meta-analysis, although the ARMD risk was found to be significantly higher in patients with vitamin D levels <50 ng/ml, a significant correlation was detected between late-stage ARMD and vitamin D levels <25 ng/ml^(16)^. In another study conducted in collaboration with the European Eye Study, including seven European countries, although the serum vitamin D level was found to be significantly lower in the neurovascular ARMD group, no association could be found in early and late dry-type ARMD^(17)^. The results of a study from Korea were consistent with our study results, wherein although a significant correlation was observed between vitamin D deficiency and earlyand late-stage ARMD, the serum vitamin D levels in patients with choroid neovascularization were found to be significantly lower than those in patients with dry-type ARMD^(18)^. In another large study from Korea, a significant association was found between late-stage ARMD and low vitamin D levels, and vitamin D was proposed to possess antineovascular and antifibrotic effects^(19)^.

In an epidemiological study conducted in the Turkish population, the rate of vitamin D deficiency was found to be 73.9%, and this rate was reported to be higher in elderly individuals^(20)^. Similarly, in our study, vitamin D levels were found to be lower than normal (<20 ng/ml) in both patients with ARMD and ageand sex-matched control patients. This finding of low vitamin D levels in both groups indicates that vitamin D deficiency is common in advanced age. Although vitamin D may be taken in the form of nutritional supplements, endogenous production is the primary source^(21,22)^. Vitamin D is primarily produced in the skin, and its production may be impaired in advanced age due to skin atrophy. Decreased sun exposure, very low body weight, obesity, nutritional deficiency, dressing style, malabsorption, and liver diseases are the other causes of vitamin D deficiency^(21)^.

There are also some studies showing that vitamin D levels are lower in smokers^(23,24)^. When we analyzed the vitamin D levels in smokers and nonsmokers, we observed lower levels in smokers. In the regression analysis, we observed that lower vitamin D levels increased the risk of developing ARMD after excluding the smoking effect. However, it is very difficult to state using our data whether smoking causes the development of ARMD by decreasing the levels of vitamin D or by some other mechanism.

Although vitamin D levels were found to be low in both groups in our study, they were significantly lower in the ARMD group than in the control group. When patients with ARMD were evaluated as early-, intermediate-, and advanced-stage, patients with the wet-type ARMD were found to have significantly lower vitamin D levels than those with the dry-type ARMD, and vitamin D deficiency was found to elevate the risk for neovascular ARMD and choroid neovascular membrane development. Supporting this finding, a previous study demonstrated that calcitriol, the active form of vitamin D, potently inhibited neovascularization^(12)^. Our study results demonstrate that the incidence of choroidal neovascularization-related diseases could increase in patients with vitamin D deficiency.

Experimental studies have revealed that cellular oxidative stress and increased inflammation, which are caused when retinal pigment cells and cone cells are exposed to H_2_O_2_, were eliminated and healthy cellular functions were regained when the cells were exposed to vitamin D. These findings suggest that vitamin D could have antioxidant and anti-inflammatory effects in the retina^(25,26)^. In a study conducted on twins with ARMD, the ARMD incidence rates were found to be significantly lower among those who received vitamin D, betaine, and methionine supplementation, and identical twins who had lower serum vitamin D levels were found to have more advanced-stage ARMD^(27)^. These findings suggest that nutrition and environmental factors play a role as much as genetic factors in the etiology of ARMD.

Studies examining whether serum vitamin D levels are influenced seasonally have yielded conflicting results. Some studies have revealed a seasonal influence, whereas the opposite result was reported in another study conducted on Asian individuals^(28,29)^. We did not evaluate vitamin D levels according to the season, which remained a limitation of our study. Nevertheless, we consider that our results are not affected because we compared the vitamin D levels between a patient group and an age-matched control group, and moreover, all blood samples were obtained at similar time points.

Another limitation of our study is that the average age of patients was approximately 70 years. In general, populations in this age group are unlikely to have normal serum vitamin D levels and be completely healthy as a control subject.

Studies investigating the effects of dietary intake of vitamin D on ARMD are limited in the literature. In a large, prospective cohort study, the risk for neovascular ARMD development was found to be lower in individuals who received vitamin D supplementation than in those who did not receive the supplementation, and no relationship was found between geographic atrophy development and vitamin D intake^(30)^.

In conclusion, low serum vitamin D levels may be a factor for the development of both ARMD and choroid neovascular membrane and subretinal fibrosis, and we suggest that vitamin D levels should be monitored at certain intervals, and if found to be low, supplementation could have a positive influence on the disease progression and visual prognosis. Further studies using larger patient populations are required.
